# Medical student attitudes towards family medicine in Spain: a statewide analysis

**DOI:** 10.1186/1471-2296-13-47

**Published:** 2012-05-29

**Authors:** Amando Martín Zurro, Josep Jiménez Villa, Antonio Monreal Hijar, Xavier Mundet Tuduri, Ángel Otero Puime, Pablo Alonso-Coello

**Affiliations:** 1 Servicio Catalán de la Salud, División de Planificación y Evaluación Operativa, Barcelona, Spain; 2 Institut Universitari d’Investigació en Atenció Primària Jordi Gol (IDIAP Jordi Gol), Gran Via 587 àtic, Barcelona, Spain; 3 Cátedra UAB-Novartis de Docencia e Investigación en Medicina de Familia, Universitat Autònoma de Barcelona, Barcelona, Spain; 4 Cátedra UNIZAR-Novartis de Docencia e Investigación en Medicina de Familia y Atención Primaria, Universidad de Zaragoza, Zaragoza, Spain; 5 Cátedra UAM-Novartis de Docencia e Investigación en Medicina de Familia y Atención Primaria, Universidad Autónoma de Madrid, Madrid, Spain; 6 Iberoamerican Cochrane Centre, CIBERESP-IIB Sant Pau, Barcelona, Spain

## Abstract

**Background:**

Family and community medicine (FM) became a recognized specialty in Spain in 1978; however, most medical schools in Spain still lack mandatory core courses in FM. In order to explore the perceptions, expectations and level of information amongst medical students in Spain in relation to FM and PC, and the training in these areas in the curriculum of the Medical Schools, a survey was developed to be administered in medical schools every two years. This article presents data from the first questionnaire administration.

**Methods:**

The study population was all first-, third-, and fifth-year students (2009–2010) in 22 participating medical schools in Spain (of 27 total). The 83-item survey had three sections: personal data, FM training, professional practice expectations, and preferences). Chi-squared test or analyses of variance were used, as appropriate.

**Results:**

We had a 41.8% response rate (n = 5299/12924); 89.8% considered the social role of FM to be essential, while only 20% believed the specialty was well respected within the medical profession. The appeal of FM increased with years of study, independent of student characteristics or medical school attended. Among third and fifth-year students, 54.6% said their specialty preferences had changed during medical school; 73.6% felt that FM specialists should teach FM courses, and 83.3% thought that FM rotations in primary care centres were useful.

**Conclusions:**

Students valued the social role of FM more highly than its scientific standing. The vast majority believe that FM training should be mandatory. Only 25% of first-year students have clear preferences for a specialization. Interest in FM increases moderately over their years of study. Working conditions in FM have decisive influence in choosing a specialty.

## Background

Family and community medicine (FM) was first included in the catalog of recognized medical specialties in Spain in 1978. Since then, the FM residency system has produced more than 35,000 specialists, most of whom are in fact practicing in the national health system [1,2]. In the past two years (2009–2010), with the development of the new educational directives set out by the European Higher Education Area (EHEA) and the Bologna Process [3], although some important advances have been made, (e.g. practical content of the curricula being more prominent and the supervision of students by tutors) the majority of the medical schools in Spain still do not have mandatory core courses in FM.

This problematic situation of FM as an academic discipline and medical specialty and of primary care (PC) as an area of professional practice is not unique to Spain [4-6]. Its limited power of attraction is the product of multiple factors: personal characteristics of students (age, sex, social class or expectations), educational environment (orientation and content of the curriculum, professors’ and fellow students’ influence, learning experience), and the perception of its professional practice (workload, possibilities for personal and professional promotion, income, opportunities for research and teaching, social prestige) [7-11].

In order to explore the perceptions, expectations and level of information medical students in Spain in relation to FM and PC, and the training in these areas in the curriculum of the Medical Schools, a survey was developed to be administered in medical schools every two years. This article presents data from the first questionnaire administration.

## Methods

The project methods have been detailed previously [12]. This article presents the results of the first administration of the survey. The protocol was approved by the Research Ethics Board at Institut Universitari d’Investigació en Atenció Primària Jordi Gol, Barcelona.

### Subjects

The target population was all students in the first, third, and fifth year of study in the 27 Spanish medical schools in 2009–2010 (approximately 15,000 students). A collaboration was established with a coordinating professor in 22 of the schools, and an investigators meeting was held with all of them to standardize data collection procedures.

### Questionnaire

The questionnaire was specifically designed for this study. An initial list of items relevant to FM was obtained from the literature review, discussed, adapted, and supplemented with new items, with the consensus of the research team. The initial version was pilot tested in May and June 2009 with 198 students in 5 medical schools. Its reliability and internal consistency was evaluated for its three sections (Cronbach’s alpha values of 0.77 [95%CI:0.61-0.88], 0.89 [95%CI:0.84-0.93] and 0.76 [95%CI: 0.65-0.85], respectively). The final version (see Additional file 1) was developed on the basis of the pilot results. The three-part questionnaire contains 70 items, plus 13 additional items specifically for third and fifth-year students:

A. Perceptions about FM (19 items): degree of agreement with statements about the social and scientific status of FM (8 items), importance of the factors that could have influenced these opinions (6 items), and feedback within the medical school about FM (5 items).

B. Preparation for FM by the medical program (26 items): opinions about the need for mandatory training in FM (5 items) and how it should be delivered (5 items), importance of contributions made by FM in various areas of the curriculum (13 items), and usefulness of PC rotations (3 items).

C. Expectations and preferences (25 items): interest in working in various areas of medicine (14 items), factors that might influence the choice of a specialty (10 items), and the degree of satisfaction anticipated if the student chose to practice FM (1 item). The questionnaires for third and fifth year students also asked about possible changes in choice of specialization since the start of medical school (8 items), participation in learning opportunities related to FM and degree of satisfaction with them (4 items), and the evolution of their level of interest in FM during the years in medical school (1 item).

Most items used a 6-point Likert scale. Questionnaires were completed anonymously. A demographic information section included medical school, year of study, sex, size of the community where pre-university studies were completed, family connections with the practice of medicine and PC, participation in voluntary service activities, and participation in university exchange programs. Printed questionnaires were designed to be automatically scanned with Teleform software.

### Data collection

Data were collected between October and December 2009. Questionnaires were distributed and gathered by each coordinating professor during educational activities. Attendance to these activities is not mandatory and therefore the presence of all students enrolled can not be guaranted. Questionnaires were completed at the end of the educational activity and delivered directly to the study coordinator in each center. A very small number of questionnaires (183) were answered over the Internet and included in the overall analysis. The students did not receive any incentive for completing the survey. All were informed in advance of the objectives and general methodology of the study.

### Data analysis

Descriptive analysis used percentages and means and standard deviations. On the 6-point Likert scales, a score equal to or greater than 4 was considered a positive assessment. Stratified analysis by medical school, year of study, and personal characteristics of the respondent were performed.

The relationship between the responses and characteristics of the medical schools was analyzed, using information gathered in a previous (June 2009) survey of the coordinating professors designed to determine the situation of FM and PC training in the participating schools [13]. The schools were classified by total number of students (1000 or more vs. <1000), availability of a specific FM course, and the existence and duration of a rotation in PC (<20 days vs. 20 days or more), and 95% confidence intervals (CI) were calculated. Comparisons used chi-square or analysis of variance, and a *P* value <0.05 was considered significant.

## Results

The total number of questionnaires returned was 5299 (2629 first-year, 1604 third-year, and 1066 fifth-year students), for an overall participation rate of 41.8% (Table 1). There were 183 Internet surveys. The missing items were excluded from the analysis, being considered invalid replies (Range = 2.0-7.1%). Of all respondents, 69.7% were women; 23.4% reported that a parent or close relative was a family physician or pediatrician in PC practice; 54.1% had a family member or friend working in these fields; 20.2% had participated in volunteer service for longer than one month; 12.7% lived on campus, worked in a Department at the Medical School, or had a scholarship; and 8.7% had participated in some type of university exchange program.

**Table 1:** Number of responses by university and number by year of study

**University**	**Numbers and rate of response**		**Year of study**		
			**First**	**Third**	**Fifth**
	**N**	% (*)	**N**	**N**	**N**
Autònoma de Barcelona	370	39.1	146	117	107
Autónoma de Madrid	359	51.6	197	99	63
Barcelona	96	19.6	27	0	69
Cantabria	78	24.0	30	27	21
Complutense de Madrid	389	38.1	183	107	99
Córdoba	322	76.8	130	131	61
Extremadura	257	74.1	127	65	65
Granada	377	48.6	169	135	73
La Laguna (Tenerife)	199	40.5	97	59	43
Las Palmas de Gran Canaria	206	83.1	131	42	33
Lleida	96	33.7	53	28	15
Málaga	57	11.8	37	20	0
Miguel Hernández (Alicante)	27	7.9	1	20	6
Murcia	262	54.1	179	76	7
Oviedo	250	74.0	113	78	59
Rovira i Virgili (Tarragona)	248	76.1	119	63	66
Salamanca	322	58.5	178	85	59
Santiago de Compostela	375	39.5	182	114	79
Sevilla	208	26.7	145	58	5
València	145	9.6	72	61	12
Valladolid	354	81.9	175	109	70
Zaragoza	302	69.9	138	110	54
**Total**	**5299**	**41.8**	**2629**	**1604**	**1066**

(*) response rate.

### Perceptions of family and community medicine

Although 89.8% of respondents considered FM to have an essential social function, fewer than 20% thought it had a high status within the medical profession, had a scientific prestige similar to other specializations, or was an interesting medical specialty from the standpoint of research (Table 2). We observed a significant increase over the curriculum in the perception of FM as an attractive career choice (36.7%, 41.7% and 50.2% in years one, three and five respectively; P <0.001). However, this trend was not observed in ther remaining items in this section of the survey. There were no differences by student or curricula characteristics.

**Table 2:** Student agreement with statements about the role of family medicine in Spain

**Do you believe that family medicine in Spain:**	Score (*)		Agreement (**)		
	**Mean**	**SD**	**%**	**95% CI**	
Has an essential social function	5.1	1.1	89.8	89.0	90.6
Is a pleasant working environment	3.9	1.3	63.6	62.3	64.9
Has a high social status	3.4	1.2	44.9	43.5	46.3
Is an attractive option	3.2	1.4	40.9	39.5	42.3
Has a high status within the medical profession	2.7	1.0	18.7	17.6	19.8
Provides a high salary in comparison with other specialties	2.7	1.3	23.4	22.2	24.6
Is an interesting specialty from a research perspective	2.6	1.1	18.0	16.9	19.1
Has a level of scientific prestige equivalent to other specialties	2.5	1.2	15.9	14.9	16.9

SD: Standard deviation; 95%CI: 95% confidence interval.

(*) Score on a Likert scale from 1 to 6 points.

(**) Score equal to or greater than 4.

The students reported that the factors exerting the most influence on their perceptions were their own experiences as a patient (61.1%) and the opinion of family or friends (57.1%) and of family doctors (53.5%). The factors that were least influential were the opinions of hospitalists, information from communications media, and their own experience in medical school, although this last factor increased in importance from 32% to 69.4% from the first to the fifth year of study (*P* <0.001). Within their medical program, 37.5% of the respondents reported hearing comments about FM, with the most favorable coming from family doctors and professors (78.4% and 67.3%, respectively). The percentage of favorable comments about FM from professors and hospitalists progressively decreased over the years of study.

### Training in family and community medicine during medical school

A large majority (87.9%) of respondents (more women (89.7%) than men (83.9%), *P* <0.001) considered there to be sufficient justification for requiring a theoretical-practical experience in FM, based on the fact that FM constitutes the majority of medical attention (92.2%), a core part of the health care system (88.3%), and the most prevalent professional option (76.6%). Only 42.7% included in their justifications the existence of specific scientific content. With respect to the methods for receiving this training, 72.8% agreed with the statement that it should be integrated into the content of related coursework, 46.9% that it should be a specific course, and 61.7% that both approaches are needed. With respect to timing, 21.2% suggested this training should be offered early (in the first or second year), compared to 34.2% preferring the third, 32.3% the fourth, and 12.3% the fifth or sixth year.

Furthermore, 73.6% agreed that FM content should be taught by family doctors and 83.3% that rotations in PC centers would be useful. Asked to assign a percentage to the appropriate amount of FM instruction, more than half (52.8%) felt that at least 25% of the practical training in medical school should be completed in FM, and 19.7% preferred 50% or more. Table 3 shows the values students assigned to FM contributions to particular areas of training. There were no differences related to the year of study or the characteristics of students or campuses. Women assigned slightly higher values.

**Table 3:** The importance of family medicine’s contributions to other areas of preparation

**Contribution**	Score (*)		Importance (**)		
	**Mean**	**SD**	**%**	**95% CI**	
Communication/Doctor-patient relationship	5.3	1.0	94.6	94.0	95.2
Clinical attention for the most common problems	5.1	1.0	93.7	93.0	94.4
Disease prevention/Health promotion	5.0	1.0	92.1	91.4	92.8
Health care across the lifespan	4.9	1.0	91.0	90.2	91.8
Family-focused health care	4.7	1.0	88.2	87.3	89.1
Biopsychosocial focus of health care	4.6	1.1	84.3	83.3	85.3
Community-focused health care	4.6	1.1	86.7	85.8	87.6
Collaboration with other sectors (education, social services or other)	4.5	1.2	80.1	79.0	81.2
Bioethics	4.2	1.3	70.4	69.1	71.7
Clinical epidemiology	4.1	1.2	68.5	67.2	69.8
Team work	4.1	1.3	66.5	65.2	67.8
Urgent care	4.0	1.5	62.9	61.6	64.2
Research	3.0	1.4	34.1	32.8	35.4

SD: Standard deviation; 95%CI: 95% confidence interval.

(*) Score on a Likert scale from 1 to 6 points.

(**) Score equal to or greater than 4.

### Expectations and preferences

Table 4 shows the students’ level of interest in various areas of specialization. Although FM received an intermediate score, interest increased significantly over time, from 41.2% to 48.7% from the first to fifth year (*P* <0.001). Women showed slightly higher levels of interest in FM than did men (46.9% vs. 40.4%; *P* = 0.003). No other variables produced relevant differences.

**Table 4:** Level of interest in various areas of specialization

**Working environment**	Score (*)		Interest (**)		
	**Mean**	**SD**	**%**	**CI 95%**	
Hospital medical specialties	4.9	1.1	88.0	87.1	88.9
Hospital surgical specialties	4.3	1.6	69.6	68.3	70.9
Pediatrics	3.7	1.7	56.1	54.7	57.5
Obstetrics and gynecology	3.3	1.6	44.6	43.2	46.0
Research	3.1	1.7	42.5	41.1	43.9
Psychiatry	3.1	1.7	40.9	39.5	42.3
Family medicine	3.1	1.5	39.4	38.0	40.8
Teaching	2.8	1.6	33.9	32.6	35.2
Dermatology	2.7	1.5	29.4	28.1	30.7
Ophthalmology	2.7	1.5	29.1	27.8	30.4
Diagnostic imaging	2.7	1.5	28.8	27.5	30.1
Laboratory	2.6	1.6	28.3	27.1	29.5
Ear, nose and throat	2.5	1.3	23.5	22.3	24.7
Preventive medicine and public health	2.4	1.4	20.7	19.6	21.8

SD: Standard deviation; 95%CI: 95% confidence interval.

(*) Score on a Likert scale from 1 to 6 points.

(**) Score equal to or greater than 4.

The importance that students gave to factors influencing their choice of specialization is shown in Table 5. The factor that the lowest number of students (55.2%) considered important was scientific prestige. Neither year of study nor campus produced relevant differences. Males gave greater importance to the level of earnings than did females (66.3% vs. 53.7%) and to scientific prestige (58.6% vs. 46.2%), and less to having close relationships with patients (79.9% vs. 85.9%).

**Table 5:** Importance of various factors in choice of specialty

**Factor**	Score (*)		Importance (**)		
	**Mean**	**SD**	**%**	**CI 95%**	
Good working conditions and quality of life	4.9	1.1	88.9	88.0	89.8
Close doctor-patient relationship	4.6	1.2	82.8	81.8	83.8
Broad spectrum of clinical problems	4.4	1.2	78.2	77.1	79.3
High level of professional dedication and commitment	4.4	1.2	77.5	76.3	78.7
Specialization requires great effort to learn	4.3	1.2	75.6	74.4	76.8
Wide range of patients of different ages	4.2	1.3	71.7	70.5	72.9
Focused on a specific spectrum of clinical problems	3.9	1.3	60.5	59.1	61.9
High level of income	3.8	1.4	60.6	59.2	62.0
Immediate outcomes of professional activities	3.7	1.3	56.1	54.7	57.5
Scientific prestige	3.7	1.5	55.2	53.8	56.6

SD: Standard deviation; 95%CI: 95% confidence interval.

(*) Score on a Likert scale from 1 to 6 points.

(**) Score equal to or greater than 4.

The average level of satisfaction that students anticipated if they ended up in family practice was 3.7 points (SD 1.3), and was 4 points or higher in 58.1% of responses. This positive assessment increased to 64.2% in fifth-year students and was slightly higher in women (62.2% vs. 57.9%; *P* = 0.05). No differences were observed on the basis of campus characteristics.

In third and fifth year students, 28% had a clear idea at the outset about the specialization they wanted to practice, while 54.6% indicated that their preferences had changed over the course of their studies. The primary reason given for this change was class content, in both theoretical and practical courses, which was considered “very important” in more than 90%. Information and opinions provided by fellow students, family, friends, and the communications media were only considered important by less than 35% of the respondents. With respect to a specific interest in FM, 37.2% indicated that this had increased over the course of their studies and 8.4% reported a decrease.

## Discussion

This nationwide study had the participation of the majority of the medical schools in Spain. Amongst the study limitations is the different reply rate of the participating schools, probably related to differences in student attendance to the activity at which the questionnaire was administered. Nevertheless, since there were not differences by campus, the school of medicine is not a relevant confounder. The lower response rate from third and fifth-year students to a survey on campus could be related to lower participation rates in campus activities in general, as well as higher rates of participation in rotations and exchange programs. Of the 27 medical schools in Spain at the time of the start of the study five did not resply to our invitation to participate. Their characteristics are not different from the rest that did, therefore, we do not consider that this has introduced any relevant bias in the results of the study. 

Students acknowledged the social importance of FM as a specialization and of the PC environment, but did not consider them to be attractive in terms of scientific-technical interest, workplace conditions, and research potential, as has been reported in other studies [14-16]. More than 75% of those surveyed think that working as a family physician would provide lower economic rewards than other areas of medicine. However, this trend was not observed in ther remaining items in this section of the survey.

The opinions of hospitalists and of FM specialists, together with the students’ own experiences, are the most important elements that determine this negative perception. This perception increases as students move through their curriculum, which might indicate that students progressively lean toward other fields of specialization and practice. The opinions of family doctors themselves are the factor that has the most influence on this trend, which might reflect their own disenchantment or unhappiness with their working conditions and professional opportunities [17].

The great majority of students (88%) agreed with the need for mandatory instruction in FM to learn about this part of the health care system, within which they will very probably practice their profession. Nevertheless, less than half thought it should be taught because of its specific scientific content.

More than 60% of those surveyed thought that a combination of integrated teaching and specific courses is the best approach to learning about FM. A majority thought that this instruction should begin in the third or fourth year, although more than 20% indicated that it should begin earlier. This view supports the curriculum design suggested by some authors [18,19] who have advocated for an early start to teaching the basic elements of FM and PC.

The students considered the essential contributions of FM training to the medical degree program to be communication skills, knowledge about disease prevention and health promotion, and attention to the most common clinical problems, followed by the biopsychosocial focus on family and community, nuclear characteristics of a comprehensive health care reinforcing the need to include them in the undergraduate curricula.

The expectations and interest level that the students demonstrated with respect to their future professional work as family physicians fall into an intermediate zone in comparison with other specialties, although the scale does not coincide with the preferences selected in the national residency program exams [20]. This suggests that other factors appear to have some influence between the end of the degree and sitting for this national exam that are difficult to identify and evaluate. In our study, in contrast with others [21,22], student interest in FM is only slightly higher among women than men (46.9% vs. 40.4%).

In agreement with other studies [23,24], anticipated working conditions have a decisive influence on the choice of a specialization, closely followed by the nature of the clinical practice itself (close doctor-patient relationships, a wide spectrum of medical conditions to treat) [25]. The income level does not appear to be considered a determining factor, in contrast to what studies have reported in countries where many students have to repay their loans that cover university costs [26,27].

Only 25% of the students who begin medical school have or think they have made a decision about the specialization they want to pursue. The most influential factors we identified that change this preference are related to the practical and theoretical content of the courses taken and the opinions and information that professors share with them. A majority (55%) of the respondents who were in their third or fifth year of study indicated that their level of interest in FM had not varied during their degree program, but 37% indicated that it had increased and 8% said it declined. In any case, if we add to these findings that only a quarter of beginning medical school students report knowing which specialization they want to practice, there is plenty of room to positively (or negatively) influence student preferences over the course of their academic program. Data from this study regarding the low status that MF and PC have from a scientific and technical view, amongst medical students, also could partially explain the low popularity it has for new graduates as a specialty choice. Despite the implementation of the Bologna process in Europe and the persistence of differences in objectives and training content in medical schools across countries, we believe that the results of this study may be of interest also from an international perspective. Administering the survey again after 2 years will enable us to assess possible changes in the overall expectations and perceptions of the student cohort. The context of these changes could be influenced by curricular modifications introduced in light of the Bologna Process or by other changes derived from a wider use of theoretical and practical FM instruction in Spain in recent years. In this regard it should be noted that in the Spanish medical school training has been mostly theoretical for many years. Only in recent times, possibly due to the influence of the Bologna process, is when there is more prominence to the practical content of learning and supervision of students by tutors. These changes should have a positive impact on enhancing MF and PC in the curriculum of Medical Schools and thus, gradually modify the results obtained in our study.

A goal of our research group is to continue this project, deepening the analysis of the perceptions and expectations of the academic world (both faculty and students) with respect to FM and PC. Our objective is to inform political and academic authorities and help them to achieve a more efficient and balanced health care system, in which FM and PC could be attractive arenas for preparation and practice for future generations of professionals.

## Conclusions

The social role, but not so much the scientific reputation, of FM is highly valued by medical students. The vast majority believe that education in FM should be mandatory. Interest in FM as a specialty increases moderately over the years of the medical degree; however, the working conditions of FM play a decisive influence in the final choice of specialty. A minority of the students who begin medical school have made a decision about the specialization they want to pursue. There is therefore room to positively (or negatively) influence student preferences over the course of their academic program.

## Abbreviations

FM, Family and community medicine; EHEA, European higher education area; PC, Primary care; SD, Standard deviation.

## Competing interests

The authors declare that they have no competing interests.

## Authors’ contributions

AMZ, JJV and PAC participated in the conception and design of the protocol and drafted a first version. All authors participated in data collection, analysis, revising the manuscript draft critically for important intellectual content and have given final approval of the final version.

## Sources of funding

This project is funded with a grant from the Instituto de Salud Carlos III, Ministerio de Sanidad, Spain (PI070975). PAC is funded by a Miguel Servet contract by the Instituto de Salud Carlos III (CP09/00137).

## Pre-publication history

The pre-publication history for this paper can be accessed here:

http://www.biomedcentral.com/1471-2296/13/47/prepub

## Supplementary Material

Additional file 1 **Student questionnaire (3rd and 5th year).** This document shows the survey completed by the students. Click here for file
