# Supplementary material for: Medical student attitudes towards family medicine in Spain: a statewide analysis
Source: BMC Fam Pract. 2012 May 29;13:47. doi: 10.1186/1471-2296-13-47 (PMC3436784; doi:10.1186/1471-2296-13-47)
Supplement: Additional file 1 — Student questionnaire (3rd and 5th year). This document shows the survey completed by the students. [file 1471-2296-13-47-S1.pdf]

1.

1. Check the name of the university you attend:

- ◯ Universitat Autònoma de Barcelona (080)
- ◯ Universidad Autónoma de Madrid (280)
- ◯ Universidad Complutense de Madrid (281)
- ◯ Universidad de Alcalá de Henares (282)
- ◯ Universitat de Barcelona (081)
- ◯ Universidad de Cantabria (390)
- ◯ Universidad de Córdoba (140)
- ◯ Universidad de Extremadura (060)
- ◯ Universidad de Granada (180)
- ◯ Universidad de La Laguna (380)
- ◯ Universidad de Las Palmas (350)
- ◯ Universitat de Lleida (250)
- ◯ Universidad de Málaga (290)
- ◯ Universidad Miguel Hernández (030)
- ◯ Universidad de Murcia (300)
- ◯ Universidad de Oviedo (330)
- ◯ Universidad de Salamanca (370)
- ◯ Universidad de Santiago de Compostela (150)
- ◯ Universidad de Sevilla (410)
- ◯ Universitat Rovira Virgili (430)
- ◯ Universidad de Valencia (460)
- ◯ Universidad de Valladolid (470)
- ◯ Universidad de Zaragoza (500)

2. What year of medical school are you in at present?

- ◯ Third
- ◯ Fifth
- ◯ Sixth

2. Perceptions about family medicine

1. Express your opinion of the following: Do you believe that in Spain family medicine:

	Totally disagree					Totally agree
Has a high level status within the medical profession	○	○	○	○	○	○
Has a high level social status	○	○	○	○	○	○
Has scientific prestige that is equivalent to other specialties	○	○	○	○	○	○
Plays an essential social role	○	○	○	○	○	○
Is an interesting specialty for research	○	○	○	○	○	○
Provides a high salary compared to other specialties	○	○	○	○	○	○
Provides a pleasant working environment	○	○	○	○	○	○
Is attractive	○	○	○	○	○	○

2. Evaluate the influence of the following factors on the opinion that you have of family medicine:

	None					Much
My personal experience as a patient	○	○	○	○	○	○
The opinion of hospital physicians about family physicians	○	○	○	○	○	○
The opinion of family physicians	○	○	○	○	○	○
The opinion of my family and/or friends	○	○	○	○	○	○
Information from the social news media	○	○	○	○	○	○
My own experience during my course of study	○	○	○	○	○	○

3. Have you heard comments about family medicine in your medical school?

NO. Go to Section B.

YES. Evaluate the comments you have heard from:

	Very negative					Very positive
Hospital physicians	○	○	○	○	○	○
Family physicians	○	○	○	○	○	○
Professors	○	○	○	○	○	○
Fellow students	○	○	○	○	○	○

3. Training in family medicine in medical school

Express your opinion of the following:

1. There are sufficient reasons

	Totally disagree					Totally agree
to justify theoretical-practical obligatory training in family medicine in medical school	○	○	○	○	○	○

2. Training in family medicine should be obligatory because:

	Totally disagree					Totally agree
It has specific scientific content	○	○	○	○	○	○
It is a common professional choice	○	○	○	○	○	○
It contributes to the improvement of healthcare	○	○	○	○	○	○
It is a central part of the health system	○	○	○	○	○	○

3. Training in family medicine should be

	Totally disagree					Totally agree
Integrated into the content of related courses	○	○	○	○	○	○
A specific course	○	○	○	○	○	○
Both ways	○	○	○	○	○	○

4. The teaching content of family medicine:

	Totally disagree					Totally agree
should always be taught by family physicians	○	○	○	○	○	○

5. In what year of medical school do you think this training should begin?

- ○ First
- ○ Second
- ○ Third
- ○ Fourth
- ○ Fifth
- ○ Sixth

6. Evaluate the importance of the following contributions of family medicine to medical school training:

	None					Much
Communication / doctor-patient relationship	◯	◯	◯	◯	◯	◯
Prevention and health promotion	◯	◯	◯	◯	◯	◯
Bio-psychosocial focus of care	◯	◯	◯	◯	◯	◯
Family focus of care	◯	◯	◯	◯	◯	◯
Community focus of care	◯	◯	◯	◯	◯	◯
Clinical care for the most frequent problems	◯	◯	◯	◯	◯	◯
Care during the life span	◯	◯	◯	◯	◯	◯
Emergencies	◯	◯	◯	◯	◯	◯
Clinical epidemiology	◯	◯	◯	◯	◯	◯
Teamwork	◯	◯	◯	◯	◯	◯
Bioethics	◯	◯	◯	◯	◯	◯
Research	◯	◯	◯	◯	◯	◯
Collaboration with other sectors (education, social, ...)	◯	◯	◯	◯	◯	◯

7. Express your opinion of the training sessions in health centres:

	None					Much
Usefulness of the sessions	◯	◯	◯	◯	◯	◯

8. During what year/s of medical school should these take place?

- ☐ First
- ☐ Second
- ☐ Third
- ☐ Fourth
- ☐ Fifth
- ☐ Sixth

9. What percentage of the total practical training in medical school do you think should be dedicated to family medicine?

- ◯ < 10%
- ◯ 10-25%
- ◯ 25-50%
- ◯ 50-75%
- ◯ 75-90%
- ◯ > 90%

4. Expectations and preferences

1. Evaluate your degree of interest in working in one of the following environments after medical school

	None					Much
Medical/hospital specialties	◯	◯	◯	◯	◯	◯
Surgical/hospital specialties	◯	◯	◯	◯	◯	◯
Family medicine	◯	◯	◯	◯	◯	◯
Paediatrícs	◯	◯	◯	◯	◯	◯
Obstetrics and gynecology	◯	◯	◯	◯	◯	◯
Psychiatry	◯	◯	◯	◯	◯	◯
Dermatology	◯	◯	◯	◯	◯	◯
Ophthalmology	◯	◯	◯	◯	◯	◯
Otorhinolaryngology	◯	◯	◯	◯	◯	◯
Preventive medicine and public health	◯	◯	◯	◯	◯	◯
Laboratory	◯	◯	◯	◯	◯	◯
Imaging diagnosis (radiology, pathoanatomy,....)	◯	◯	◯	◯	◯	◯
Research	◯	◯	◯	◯	◯	◯
Teaching	◯	◯	◯	◯	◯	◯

2. Evaluate the importance you will give to the following characteristics in your selection of a specialty after you finish medical school

	None					Much
Covers an ample spectrum of clinical problems	○	○	○	○	○	○
Allows for care of a wide range of patients of different ages	○	○	○	○	○	○
Centers on a more concrete spectrum of clinical problems	○	○	○	○	○	○
Allows for the immediate visualization of the results of professional activity	○	○	○	○	○	○
Has scientific prestige	○	○	○	○	○	○
Requires a high level of professional dedication and commitment	○	○	○	○	○	○
Training in a specialty requires much effort	○	○	○	○	○	○
Provides a high earning capability	○	○	○	○	○	○
Allows for a close relationship with patients	○	○	○	○	○	○
Allows for good working conditions and quality of life	○	○	○	○	○	○

3. If in future you should work as a family physician,

	Very low					Very high
what would your level of satisfaction be?	○	○	○	○	○	○

4. When you started in medical school, had you made a clear decision about the specialty you wanted to follow?

○ No

○ Yes

5. Have you changed your specialty preferences as you've gone through medical school?

No. Go to the next question

YES. Evaluate the importance of the following factors in your change of preferences

	None					Much
Opinions and information from professionals	○	○	○	○	○	○
Opinions and information from professors	○	○	○	○	○	○
Opinions and information from fellow students	○	○	○	○	○	○
Opinions and information from relatives and friends	○	○	○	○	○	○
Information from the social media	○	○	○	○	○	○
Theoretical content of the courses	○	○	○	○	○	○
Practical content of the courses	○	○	○	○	○	○

6. Have you had courses or seminars related to family medicine?

NO. go to the next question

YES. Evaluate your satisfaction

	None					Much
with this activity	○	○	○	○	○	○

7. Have you had practice sessions/practical activity in family medicine?

NO. go to the next question

YES. Evaluate your satisfaction

	None					Much
with this activity	○	○	○	○	○	○

8. During your years of medical school, would you say your interest in family medicine:

- ○ Has increased
- ○ Has decreased
- ○ Has not changed

5. Personal information

1. Gender

- Male
- Female

2. What is the population of the area where you went to school (before medical school)?

- < 10.000 inhabitants
- 10.000 - 300.000 inhabitants
- > 300.000 inhabitants

3. Is one of your parents or relatives a family physician / general practitioner / country doctor / paediatrician?

- No
- Yes

4. Is one of your parents or close relatives a hospital specialist physician?

- No
- Yes

5. Do you have a relative or friend working in primary care / health centre (nurse, midwife, clinical assistant, secretary)?

- No
- Yes

6. Does either of your parents or tutors hold a university degree?

- No
- Yes

7. Have you ever done voluntary work in an organization for periods longer than one month?

- No
- Yes

8. Do you normally attend one of the medical school departments as an intern, collaborator or scholarship student?

- No
- Yes

9. Have you taken part in any university interchange program, i.e., Erasmus?

- ◯ No
- ◯ Yes

10. If you answered yes to the previous question, during what study year/s?

- ☐ First
- ☐ Second
- ☐ Third
- ☐ Fourth
- ☐ Fifth
- ☐ Sixth

6. FIS Project

Thank you for your collaboration.
